# Weak association between arterial hypertension and overactive bladder baseline symptoms and treatment responses

**DOI:** 10.3389/fphar.2022.1081074

**Published:** 2022-12-13

**Authors:** Martin C. Michel, Uwe Heemann, Jean J. M. C. H. de la Rosette

**Affiliations:** ^1^ Department of Pharmacology, Johannes Gutenberg University, Mainz, Germany; ^2^ Department of Medicine, University Medical Center, Munich, Germany; ^3^ Department of Urology, Medipol Mega University Hospital, Istanbul Medipol University, Istanbul, Turkey

**Keywords:** overactive bladder syndrome, arterial hypertension, non-interventional study, pathophysiology, solifenacin, treatment

## Abstract

While animal studies have suggested an association between the presence of hypertension and the presence and/or severity of overactive bladder syndrome (OAB) symptoms, little clinical data is available. We have conducted a pre-specified secondary analysis of a non-interventional study involving 4450 OAB patients being treated with solifenacin to explore the existence of an association between OAB and hypertension using three parallel and overlapping definitions of hypertension to enhance robustness of analysis. Regardless of definition, patients with hypertension were older and had greater OAB symptom severity in univariate analyses. In multiple regression models including age as explanatory covariate, most relationships held up but effect sizes of concomitant hypertension on OAB severity were small (odds ratios <1.35 in all cases) and were deemed to be unlikely of clinical relevance. % Changes in symptom severity were somewhat smaller in univariate analysis, but effect sizes were small. We conclude that OAB and arterial hypertension are associated but effect sizes are too small to justify adaptation of clinical practice for OAB patients with concomitant hypertension.

## 1 Introduction

Chronic pelvic hypoperfusion has been proposed as a possible cause of lower urinary tract symptoms (LUTS) including overactive bladder syndrome (OAB) and male LUTS attributed to benign prostatic hyperplasia (BPH); atherosclerosis of pelvic vessels is considered as the main reason for the hypoperfusion (Michel et al., 2015; Thurmond et al., 2016). Established key risk factors for the development of atherosclerosis are metabolic syndrome, diabetes, and arterial hypertension (HT) (Libby, 2021). As diabetes, hypertension, OAB and male LUTS attributed to BPH are frequent in the population and exhibit increasing prevalence with age, it can be expected that they occur in parallel based on chance alone. However, several studies indicate that the extent of association of male LUTS attributed to BPH, and concomitant comorbidities linked to an elevated risk of atherosclerosis exceeds what is expected based on chance alone.

Associations of diabetes and HT were reported for LUTS in general, i.e., not differentiating between OAB, BPH or other specific diagnoses (Zhang et al., 2015). More specifically, large datasets have indicated that diabetes (Michel et al., 2000; Rohrmann et al., 2005; Gupta et al., 2006; Martin et al., 2011) or HT (Joseph et al., 2003; Michel et al., 2004; Rohrmann et al., 2005; Ponholzer et al., 2006) are associated with greater baseline symptoms in male LUTS attributed to BPH, although that was not confirmed in all studies (Gupta et al., 2006; Martin et al., 2011). The presence of diabetes (Fu et al., 2016) or HT was also reported to be associated with a greater progression of male LUTS (Marshall et al., 2014; Fu et al., 2016). Interestingly, both HT and male LUTS can be treated with α_1_-adrenoceptor antagonists, although they are no longer considered a first-line treatment for HT because other anti-hypertensive drug classes exhibit a better benefit/risk profile in this indication (Allhat Officers, 2000).

Much less clinical data has been reported for a possible association between HT and OAB; the only major study coming from a general health screening program and using the International Prostate Symptom Score as a tool to quantify LUTS but no specific assessment of OAB symptoms (Ponholzer et al., 2006). On the other hand, various studies in experimental animals link atherosclerosis in general (Nomiya et al., 2012; Bschleipfer et al., 2015) and its key risk factor HT to an OAB-like phenotype: Spontaneously hypertensive rats, one of the most frequently used animal models of HT, exhibit many features of OAB patients including detrusor overactivity and frequent micturitions and are considered to be an animal model of both, HT and OAB (Persson et al., 1998; Spitsbergen et al., 1998; Steers et al., 1999). In cross-breeding studies the presence of HT and OAB-like phenotype co-segregate (Clemow et al., 1999), indicating a genetic link between both conditions in rats. Moreover, animal models of acquired HT also have been reported to be associated with bladder dysfunction. E.g., chronic inhibition of nitric oxide synthase increases blood pressure and has been shown to shift the balance of rat bladder tone towards a more contractile state (Monica et al., 2008). Similarly, long-term fructose feeding of rats induced bladder overactivity (Lee et al., 2008), and the bladder of renal hypertensive rats exhibits enhanced contraction to muscarinic agonists and reduced relaxation to β-adrenoceptor agonists (Ramos-Filho et al., 2011).

Therefore, we have explored a possible association between OAB symptoms and the presence of HT using the baseline data of an observational study comprising 4450 OAB patients (Michel et al., 2008). Additionally, a possible impact of the presence of HT on the therapeutic response to a muscarinic receptor antagonist has been explored in these OAB patients as a secondary aim.

## 2 Patients and methods

### 2.1 Study population

This manuscript describes an investigator-initiated, pre-planned, secondary analysis of an open-label, non-interventional study into the safety and efficacy of solifenacin in patients with OAB, which was performed as part of the post-marketing surveillance in Germany (Michel et al., 2008). Thus, no specific inclusion and exclusion criteria were applied other than a minimum age of 18 years and the recommendations from the Summary of Product Characteristics for solifenacin. Of note men with possible concomitant BPH were not formally excluded from participation. Rather the participating 1316 office-based urologists were asked to systematically record their observation for patients receiving solifenacin based upon their medical judgment (*n* = 4450, 83.5% women). Based upon the package insert, recommended solifenacin doses were 5 and 10 mg q. d. The planned duration of treatment was 12 weeks. Other prespecified analyses from the same database have been reported (Witte et al., 2009; Michel et al., 2011). Applicable German laws and regulations neither required nor recommended ethical committee approval at the time the study was performed.

### 2.2 Patient evaluation

The present analysis is based upon the office visit prior to and at the end of treatment, hereafter referred to as baseline and treatment data, respectively. In cases of premature study discontinuation, the data from the last available visit were used in a last-observation-carried-forward manner. At both visits, a range of assessments related to OAB severity were made. They included episode frequency of the OAB symptoms urgency, incontinence, voiding frequency, and nocturia (Abrams et al., 2002), pad use and mean micturition volume. Additionally, blood pressure was measured (at least two measurements in sitting position at least 5 min apart). At the baseline visit; the case record form explicitly asked for arterial hypertension as comorbidity as well as for all existing concomitant medications. Among the latter diuretics, β-adrenoceptor antagonists, Ca^2+^-channel blockers, angiotensin converting enzyme inhibitors and angiotensin receptor antagonists were considered as anti-hypertensive drugs, although they may have been prescribed for other indications.

### 2.3 Data analysis

In line with our previous studies on associations between BPH-attributed LUTS and HT (Michel et al., 2004), we operationally defined the presence of HT in three parallel but overlapping ways to enhance the robustness of our analysis. Firstly, we have defined patients as hypertensive if they had a measured diastolic blood pressure (P_diast_) > 90 mm Hg (“measured HT”) during the baseline office visit. Second, we defined patients as hypertensive if the urologist had recorded this as a known comorbidity in response to a specific question on the case record form (“diagnosed HT”). Finally, we defined patients as hypertensive if they were receiving at least one medication that is typically prescribed for the treatment of HT, e.g., diuretics, β-adrenoceptor antagonists, Ca^2+^-channel blockers, angiotensin converting enzyme inhibitors or angiotensin receptor antagonists (“treated HT”). As the number of prescribed anti-hypertensive medications can be an indication of disease severity, we have subdivided the latter group into patients with 1, 2 or >2 concomitant anti-hypertensive medications. In comparison with all three operational definitions of HT, normotensive patients were defined as those with a P_diast_ ≤90 mm Hg (mean 79 ± 7 mm Hg), no recorded diagnosis of HT, and not reporting any concomitant treatment with anti-hypertensive drugs.

Data handling was performed by Medidata (Konstanz, Germany), a contract research organization, based on a statistical analysis plan developed by the authors. Note that in some cases reported percentages do not add up to 100% due to missing data (always <10%, much less in most cases). Baseline data are presented as means ± SD of absolute values of n patients. In line with previous similar analyses (Michel et al., 2007), treatment data are presented as % changes for OAB symptoms and pad use because % changes are closer to a normal distribution than absolute changes (Amiri et al., 2020); changes for the voided volume were expressed as absolute differences. Additionally, multiple logistic regression models were applied to the baseline data to determine the odds ratio of having a greater number of micturitions, urgency, nocturia or urgency incontinence episodes in HT vs normotensive subjects; age was used as a co-explanatory variable in these models. All statistical analysis was performed with the SAS program package (version 9.1.3). In line with the exploratory character of our analyses and with recent recommendations from leading statisticians (Amrhein et al., 2019; Michel et al., 2020), we did not calculate *p*-values but rather focused on effect sizes with their 95% confidence intervals (CI).

## 3 Results

### 3.1 Baseline data

Patients with measured, diagnosed or treated HT were an average of 9–11 years older than normotensive subjects (Table 1). A greater number of concomitant anti-hypertensive agents was also associated with higher age within the treated HT group (Table 1). HT patients, irrespective of definition of HT, also exhibited a greater number of incontinence, micturition, and nocturia episodes and greater pad use. In contrast, number of urgency episodes and mean voided volume did not differ consistently between HT and normotensive patients.

**TABLE 1:** Baseline parameters in normotensive and hypertensive patients.

Group	N	Age	Urgency	Incontinence	Micturitions	Nocturia	Pads	Voided volume
Normotensives	2194	58 ± 13	9.4 ± 6.1	4.3 ± 3.3	13.2 ± 4.8	3.6 ± 2.0	4.1 ± 3.0	86.4 ± 51.4
Measured HT	453	67 ± 11	9.5 ± 5.5	5.0 ± 4.2	13.6 ± 4.7	3.9 ± 1.7	4.7 ± 3.5	89.9 ± 59.0
Diagnosed HT	1857	69 ± 10	9.0 ± 5.9	4.9 ± 4.0	13.4 ± 5.0	4.0 ± 1.9	4.8 ± 3.3	78.5 ± 57.0
Treated HT all	1780	69 ± 10	8.9 ± 5.9	4.9 ± 4.0	13.4 ± 5.0	4.1 ± 1.9	4.8 ± 3.2	77.5 ± 56.2
Treated HT n = 1	1066	68 ± 10	8.7 ± 5.4	4.7 ± 3.7	13.3 ± 4.6	4.0 ± 1.9	4.7 ± 3.1	76.2 ± 46.6
Treated HT n = 2	470	71 ± 10	9.1 ± 6.5	5.0 ± 4.3	13.6 ± 5.7	4.2 ± 1.9	4.9 ± 3.3	73.9 ± 49.7
Treated HT n > 2	244	73 ± 10	9.3 ± 6.8	5.7 ± 4.9	13.6 ± 5.0	4.2 ± 1.9	5.0 ± 3.5	90.3 ± 94.0

N is the number of patients in the respective group, whereas n in treated HT, refers to number of antihypertensive drugs. All data are means ± SD, and are given as episodes per 24 h except for age (years) and voided volume/void (ml).

Therefore, statistical comparisons between groups for OAB-related parameters were performed using multiple logistic regression models with age as a covariate. The presence of HT was consistently associated with a numerically positive odds ratio for having more incontinence, micturition, and nocturia episodes, but the CI did not exclude unity in four of nine models (Table 2). In contrast, measured HT was associated with a greater odds ratio for having more urgency, whereas diagnosed and treated HT were associated with a smaller odds ratio. However, all odds ratios, whether significantly different from one or not, deviated numerically from one by a small margin (0.862–1.345).

**TABLE 2:** Multiple logistic regression analysis for measured, diagnosed or treated HT to be associated with a greater episode frequency of OAB symptoms. Data are shown as odds ratio with 95% confidence interval for a greater number of OAB symptom episodes/24 h as a function of HT. Odds ratios are relative to the normotensive patient group, and age has been used as a co-explanatory variable in the models. Confidence interval excluding one indicate a statistically significant difference as compared to the reference group (normotensive patients).

	Urgency	Incontinence	Micturitions	Nocturia
Measured HT	1.195 (1.006–1.419)	1.164 (0.973–1.392)	1.223 (1.030–1.45)	1.102 (0.925–1.313)
Diagnosed HT	0.886 (0.790–0.993)	1.162 (1.030–1.312)	1.087 (0.970–1.218)	1.323 (1.178–1.487)
Treated HT	0.862 (0.768–0.968)	1.150 (1.018–1.299)	1.102 (0.982–1.236)	1.345 (1.196–1.513)

### 3.2 Treatment responses

HT patients were numerically more often prescribed the 10 mg dose of solifenacin at study end by their urologists, and this was consistently found for all three HT definitions (Table 3). As described earlier (Witte et al., 2009), treatment-associated improvements of number of urgency (−63%), incontinence (−73%), micturition (−39%), and nocturia episodes (−59%), of pad use (−59%) and mean voided volume (+67%) were substantial. Irrespective of applied definition, HT patients exhibited numerically smaller % improvements in number of urgency, incontinence, micturition, and nocturia episodes, in pad used and in increase of voided volume (Table 3). Such differences were not consistently observed based on number of concomitant anti-hypertensive medications within the treated HT group. Moreover, the effect sizes were only small relative to the overall % improvement in all cases.

**TABLE 3:** Treatment responses in normotensive and hypertensive patients.

Group	5/10 mg dose	Urgency	Incontinence	Micturitions	Nocturia	Pads	Volume, ml
Normotensives	75.1/17.2	−66 ± 33	−77 ± 38	−40 ± 17	−62 ± 29	−62 ± 34	+70 ± 169
Measured HT	67.1/23.8	−60 ± 27	−71 ± 41	−37 ± 17	−54 ± 25	−55 ± 32	+55 ± 118
Diagnosed HT	70.6/21.3	−59 ± 30	−70 ± 36	−39 ± 17	−57 ± 25	−55 ± 32	+63 ± 136
Treated HT all	70.9/21.2	−60 ± 29	−71 ± 34	−39 ± 17	−57 ± 25	−56 ± 32	+65 ± 138
Treated HT n = 1	74.2/19.0	−60 ± 28	−71 ± 33	−39 ± 17	−57 ± 26	−57 ± 31	+60 ± 100
Treated HT n = 2	65.3/25.3	−61 ± 32	−71 ± 37	−40 ± 17	−58 ± 24	−55 ± 34	+78 ± 196
Treated HT n > 2	67.2/23.0	−58 ± 26	−70 ± 32	−37 ± 17	−52 ± 24	−52 ± 31	+62 ± 162

n in treated HT, refers to number of antihypertensive drugs. All data are means ± SD, and shown as % reduction in the respective parameter except for drug dose at study end (shown as % of patients on 5 and 10 mg solifenacin).

## 4 Discussion

In humans, LUTS attributed to BPH were associated with HT to a greater extent than expected based on chance alone in most studies (Joseph et al., 2003; Michel et al., 2004; Rohrmann et al., 2005; Ponholzer et al., 2006). While animal models of HT consistently show an OAB-like phenotype (Persson et al., 1998; Spitsbergen et al., 1998; Clemow et al., 1999; Steers et al., 1999; Monica et al., 2008; Ramos-Filho et al., 2011), only little data is available to test whether such associations also exist in patients. Given possible cardiovascular effects of agents used to treat OAB (Brodde and Michel, 1999; Andersson and Olshansky, 2007; Michel and Gravas, 2016), it appears important to know whether OAB and HT are linked in humans and how this may affect therapeutic responses to OAB medications.

### 4.1 Critique of methods

The present analysis is based upon a non-interventional study performed by office-based, board-certified urologists in Germany (Michel et al., 2008). Exploration of associations between OAB and HT was an investigator-initiated, pre-specified secondary study aim. To optimize comparability with previous findings on associations between HT and LUTS attributed to BPH (Michel et al., 2004), the present analyses are similar to those used in our previous analyses. Based on the non-interventional character of the underlying study, participating physicians did not receive instructions on how to diagnose comorbidities, e.g., those in line with the definitions provided by the World Health Organization. Moreover, the diagnosis of HT is not the core business of urologists. Therefore, we have applied three concomitant and overlapping definitions of HT to increase the robustness of our analysis. These definitions were the same as in our previous analysis in a BPH population (Michel et al., 2004).

Use of data from a non-interventional study provides advantages and limitations. Firstly, due to lack of strict inclusion and exclusion criteria other than those in the Summary of Product Characteristics, the resulting data are less biased and more representative for the general population compared to those from randomized controlled trials with their typically long list of inclusion and exclusion criteria. Second, our study is based on patients seeking treatment for OAB symptoms, which may have caused some degree of bias as compared to a population-based study. Third, based on involving only board-certified urologists as investigators we can assume a more reliable diagnosis of OAB and quantification of OAB symptoms than could be expected from a population-based study. Fourth, relying on data obtained by urologists, measurement of blood pressure and capturing of comorbidities and comedications may have been less stringent than e.g., by cardiologists or nephrologists. We have no data on duration of HT and used number of anti-hypertensive medications as a proxy for the severity of HT. Finally, a non-interventional study as ours involved neither a control group nor a placebo-controlled run-in phase. This implies that the observed treatment-associated improvements of OAB symptoms should not be taken as absolute values but rather for a relative comparison of normotensive and HT patients. However, the efficacy of solifenacin in the treatment of OAB symptoms has been documented in several previous placebo-controlled studies (Chapple et al., 2008) and their meta-analyses (Wang et al., 2014; Reynolds et al., 2015).

It is a strength of the present data that all analyses are based on a pre-specified study protocol, i.e., no *post hoc* analyses were performed. Knowing that both OAB and HT are age-related conditions, we had expected that OAB patients with HT would be older than normotensive subjects. Therefore, the analysis of the baseline data related to a pathophysiological association between OAB and HT were based on multiple logistic regression models including age as a covariate. While our analyses on the association between OAB and HT based on baseline data should be applicable universally, our findings related to treatment of OAB are limited to solifenacin because no other drug has been studied here. However, we have no reason to assume that different findings would be obtained if other drugs using the same mechanism of action (i.e., antagonism of muscarinic receptors) were applied.

### 4.2 Baseline data

The clinical evidence for a relationship between OAB and HT is limited. To the best of our knowledge, the only major study comes from a general health screening program involving 2536 men and women, of whom 27% and 31%, respectively exhibited LUTS (Ponholzer et al., 2006). That study had quantified LUTS in both genders by the International Prostate Symptom Score, did not differentiate possible causes of LUTS, made no specific assessment of OAB symptoms, and did not take treatment status of hypertensive subjects into account. Under these conditions, it did not detect a statistically significant association between LUTS and HT. Therefore, our analysis appears to be the first clinical study exploring an association between OAB and HT as a pre-specified study aim.

Our descriptive analyses show that measured HT, diagnosed HT and treated HT were consistently associated with a numerically greater age and worse OAB symptoms (more incontinence and nocturia episodes, greater urinary frequency, greater pad use and lower voided volume (Table 1). Out of the 21 comparisons, only few did not show this trend (urgency in diagnosed and in treated HT, voided volume in measured HT). Moreover, more severe hypertension as indicated by using of more anti-hypertensive medications exhibited a ‘dose-response’ relationship with signs and symptoms of OAB, except for voided volume in the group with more than two anti-hypertensive medications.

Particularly, the age effect had been expected based on our previous similar analyses in a BPH population (Michel et al., 2004). Therefore, our pre-specified study protocol had included a multiple logistic regression analysis using age as explanatory co-variable. This yielded mixed results: the odds ratio to have more incontinence and nocturia episodes and more micturitions was greater than unity but the corresponding CI included unity in four of nine analyses. Moreover, the odds ratio for having more urgency episodes was greater than unity only for measured HT, whereas it (and its CI) was smaller than unity for diagnosed HT and treated HT (Table 2). Perhaps more importantly, all odds ratios (whether CI included unity or not) deviated only to a small extent from unity, indicating a weak if any association between OAB and HT. These findings are compatible with those of a previous study not specifically focusing on OAB symptoms (Ponholzer et al., 2006). While the data in our previous BPH study had more consistently indicated an association between International Prostate Symptom Score and maximum urine flow rate as indicators of BPH severity on the one hand and measured HT, diagnosed HT, and treated HT on the other hand, effect sizes in that study were also small (Michel et al., 2004).

This raises the question whether the present data support or refute the hypoperfusion hypothesis of LUTS in general and OAB in particular (Michel et al., 2015; Thurmond et al., 2016). We feel that they tend to support the hypothesis. However, the extrapolation from HT as risk factor for atherosclerosis to chronic pelvic hypoperfusion may be too indirect to lead to strong associations between the presence of HT and OAB severity.

### 4.3 Treatment data

To the best of our knowledge, the impact of HT on treatment responses in OAB patients has not been explored in the past. Therefore, such analysis was an exploratory secondary aim of our study. Interestingly, HT patients were somewhat more likely to receive the 10 mg dose of solifenacin than normotensive patients. This was true for all definitions of HT and for all numbers of anti-hypertensive comedications (19.0–25.3% on 10 mg dose) as compared to normotensives (17.2% on 10 mg dose).

It has recently been shown that improvements of OAB symptoms upon treatment do not exhibit a normal distribution, which makes reporting of mean changes suboptimal and potentially misleading (Amiri et al., 2020). While normality tests in large cohorts also detected a statistically significant deviation from unity for % changes of OAB symptoms, this deviation was much smaller than for absolute changes and deemed to be of limited clinical relevance. Therefore, our exploratory analysis of treatment responses was based on % changes.

In univariate, descriptive analysis, % changes of OAB symptoms in all HT groups were numerically smaller than in normotensive patients (Table 3). However, these differences were small relative to the overall improvement. Considering that the smaller % reductions in OAB symptoms came on a background of greater baseline symptoms (Table 1), the overall impact of concomitant HT probably is of little clinical relevance. A similar conclusion had been drawn for the impact of concomitant diabetes on OAB treatment outcomes with a different muscarinic receptor antagonist (Schneider et al., 2013). Thus, these data indicate similar improvements of OAB symptoms in normotensive and hypertensive patients within the ranges that are considered to be of clinical relevance.

## 5 Conclusion

We conclude that an association between the presence of OAB and HT appears to exist to a greater extent than based on chance alone for two age-related conditions; however, the strength of association is weak. These human data contrast those from various animal models (Persson et al., 1998; Spitsbergen et al., 1998; Clemow et al., 1999; Steers et al., 1999; Monica et al., 2008; Ramos-Filho et al., 2011). While we lack a mechanistic explanation for such differences between experimental and clinical data, two not mutually excluding possibilities come to mind. Firstly, human populations, particularly if sampled from real-world evidence studies, are considerably more heterogeneous than animal models of disease. Even if an effect exists, its relevance relative to many other factors may be smaller in clinical studies than in animal models. Second, human hypertension can have many causes, whereas animal models typically are mono-causal. Therefore, such animal models have benefits to identify possible pathophysiological mechanisms but may have modest translational value (Erdogan and Michel, 2020). Our pathophysiological baseline data and treatment outcome findings indicate that the effect sizes presently found are most likely of insufficient magnitude to warrant implications for clinical patient management and probably too small to have implications for the clinical management of OAB or HT patients.

## Data Availability

The data analyzed in this study is subject to the following licenses/restrictions: The data are owned by Astellas Pharma GmbH. All analyses reported in the manuscript are available from the authors to qualified investigators upon reasonable request. Requests to access these datasets should be directed to marmiche@uni-mainz.de.
